# Invasive diagnostic and therapeutic measures are unnecessary in patients with symptomatic van Neck–Odelberg disease (ischiopubic synchondrosis): a retrospective single-center study of 21 patients with median follow-up of 5 years

**DOI:** 10.1080/17453674.2021.1882237

**Published:** 2021-02-04

**Authors:** Kristian Nikolaus Schneider, Lukas Peter Lampe, Georg Gosheger, Christoph Theil, Max Masthoff, Robert Rödl, Björn Vogt, Dimosthenis Andreou

**Affiliations:** a Department of Orthopedics and Tumor Orthopedics; University Hospital of Münster, Münster;; b Department of Radiology; University Hospital of Münster, Münster, Germany

## Abstract

Background and purpose — Van Neck–Odelberg disease (VND) is a self-limiting skeletal phenomenon characterized by a symptomatic or asymptomatic uni- or bilateral overgrowth of the pre-pubescent ischiopubic synchondrosis. It is frequently misinterpreted as a neoplastic, traumatic, or infectious process, often resulting in excessive diagnostic and therapeutic measures. This study assessed the demographic, clinical, and radiographic features of the condition and analyzed diagnostic and therapeutic pathways in a large single-center cohort.

Patients and methods — We retrospectively analyzed 21 consecutive patients (13 male) with a median age of 10 years (IQR 8–13) and a median follow-up of 5 years (IQR 42–94 months), who were diagnosed at our department between 1995 and 2019.

Results — VND was unilateral in 17 cases and bilateral in 4 cases. Initial referral diagnoses included suspected primary bone tumor (n = 9), fracture (n = 3), osteomyelitis (n = 2), and metastasis (n = 1). The referral diagnosis was more likely to be VND in asymptomatic than symptomatic patients (4/6 vs. 2/15). More MRI scans were performed in unilateral than bilateral VND (median 2 vs. 0). All 15 symptomatic patients underwent nonoperative treatment and reported a resolution of symptoms and return to physical activity after a median time of 5 months (IQR 0–6).

Interpretation — By understanding the physiological course of VND during skeletal maturation, unnecessary diagnostic and therapeutic measures can be avoided and uncertainty and anxiety amongst affected patients, their families, and treating physicians can be minimized.

Van Neck–Odelberg disease (VND) is a self-limiting skeletal phenomenon characterized by an asymptomatic or symptomatic uni- or bilateral overgrowth of the pre-pubescent junction between the inferior pubic ramus and ischium, which can be seen on radiographs during skeletal maturation (Figure 1) (Herneth et al. 2004, Wait et al. 2011, Mixa et al. 2017). The condition was first described in the 1920s by Odelberg and Van Neck, who classified it as a “disease” (Odelberg 1923, van Neck 1924). Today, however, VND is considered a physiological normal variant of the ischiopubic synchondrosis (IPS) that is usually obliterated between late childhood and early adolescence by bony fusion or synostosis (Herneth et al. 2000, Mixa et al. 2017).

**Figure 1.**
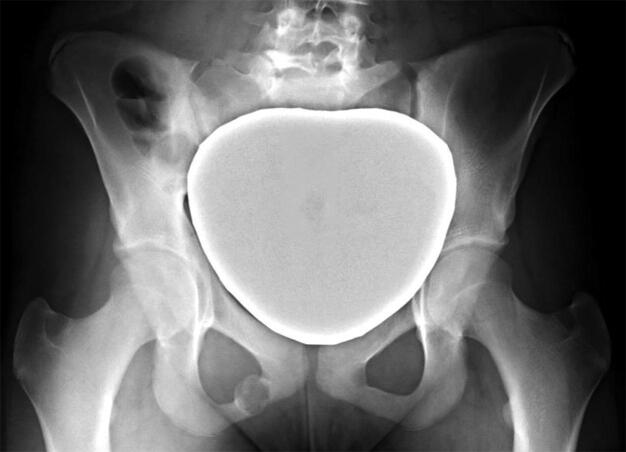
Typical enlargement of the IPS in a 14-year-old female patient with right-sided VND.

Despite VND being considered a normal variant, unilateral radiographic changes of the IPS are frequently misinterpreted as neoplastic, traumatic, or infectious processes (Herneth et al. 2000, Wait et al. 2011). Particularly in symptomatic patients, this often results in excessive, unnecessary, invasive, and costly diagnostic measures causing uncertainty and anxiety amongst patients and their families, as well as treating physicians (Herneth et al. 2000, Wait et al. 2011).

There are several case reports on VND in the literature; however, there are only 1 small series of 10 cases and 1 systematic review of 29 patients available, providing only limited data on demographics, possible diagnostic and therapeutic pathways, and patient outcome (Wait et al. 2011, Mixa et al. 2017). We therefore conducted this study to assess demographic, clinical, and radiographic features of VND, analyze the course of the disease, and evaluate the functional follow-up in a large single-center cohort. Based on these data, we additionally developed a standardized algorithm to help minimize the use of unnecessary diagnostic measures and to simplify diagnosis.

## Patients and methods

Data on patients who were diagnosed with VND at our department between 1995 and 2019 were retrieved from our hospital information system. 21 patients (13 male) were identified and included in the study. Pertinent data regarding diagnosis, clinical and radiographic features, as well as patient treatment and follow-up were retrospectively obtained from patients’ records.

**Figure 2.**
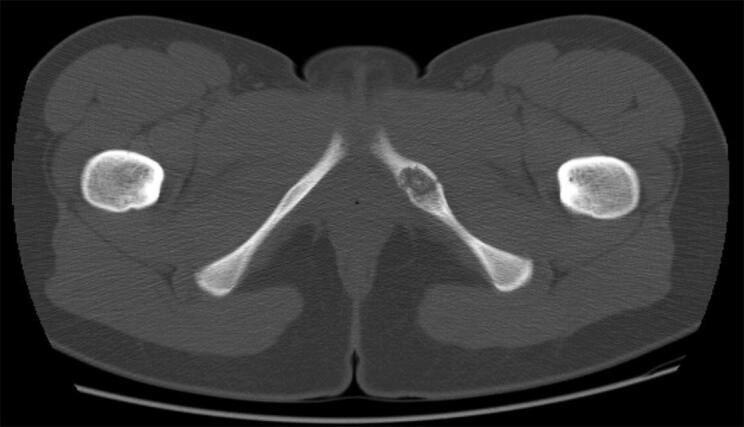
CT scan showing enlargement of the left IPS in a 16-year-old female patient with VND.

**Figure 3.**
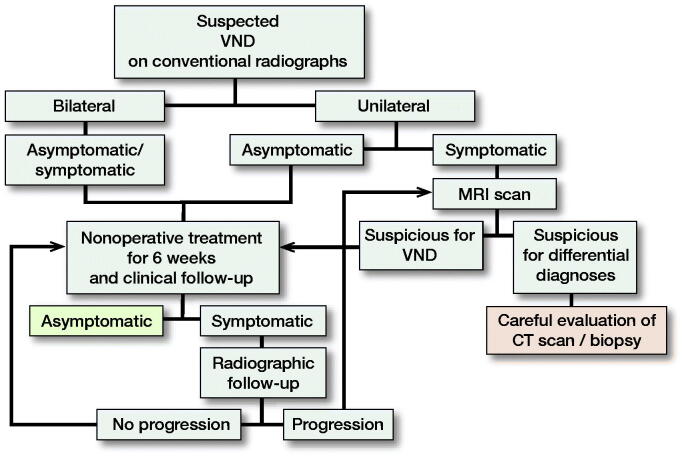
Standardized algorithm in our institute for diagnosis and treatment of VND.

All patients were consulted by specialized tumor or pediatric orthopedic surgeons. Patients presenting with only an MRI underwent additional radiographic imaging to confirm diagnosis, whereas patients presenting with radiographs and/or computed tomography (CT) scans did not undergo additional MRI if the conventional radiographic findings were typical of VND (Figure 2). Symptomatic patients were treated with analgesics and physical therapy, and were advised to refrain from sports and painful physical activities for 6 weeks. Symptomatic patients underwent a clinical and radiographic follow-up examination after 3–4 months to exclude a relevant growth. Further consultations were recommended only in patients with persisting or new symptoms (Figure 3).

### Statistics

Medians with ranges were calculated for non-normally distributed data. Contingency tables were analyzed using the chi-square test. Non-parametric analyses were performed with the Mann–Whitney U-test. The distribution of dichotomous variables was evaluated with a 1-sample binominal test. All p-values were 2-sided; a p-value < 0.05 was considered significant. Statistical calculations were performed with SPSS Version 25.0 (IBM Corp, Armonk, NY, USA).

### Ethics, funding, and potential conflicts of interest

The study was approved by the local ethics committee (Ethik-Kommission der Ärztekammer Westfalen-Lippe, No. 2019-553-f-S) and performed in accordance with the Declaration of Helsinki. We acknowledge support from the Open Access Publication Fund of the University of Münster. The authors have no conflicts of interest to report.

## Results

The median age at diagnosis in our cohort was 10 years (IQR 8–13), and similar between male and female patients (median 9 vs. 11 years). The median follow-up was 5 years (IQR 42–94 months). 17 patients presented with a uni- and 4 patients with a bilateral VND. 15 patients presented with local complaints, consisting of groin pain (n = 10), gluteal pain (n = 2), upper leg pain (n = 2), and hip pain (n = 1). VND was an incidental finding in the remaining 6 patients who underwent imaging due to Legg–Calvé–Perthes disease (n = 2), and 1 case each for coxitis fugax, staging for Ewing’s sarcoma of the right ulna, and persistent contralateral knee pain and trauma. Initial referral diagnoses by local doctors to the outpatient clinic of our tertiary hospital included suspected primary bone tumors (n = 9), fractures (n = 3), osteomyelitis (n = 2), and bone metastasis (n = 1). In the remaining 6 patients the suspected diagnosis was VND. The suspected diagnosis by the referring physicians was more likely to be VND in asymptomatic than in symptomatic patients (4/6 vs. 2/15, p = 0.02).

55 conventional radiographs, 37 MRI scans, and 9 CT scans were performed in our cohort. The 2 patients with Legg–Calvé–Perthes disease, who had bilateral VND, underwent 8 radiographic examinations each to monitor the development of the disease and the containment of the femoral head. Among the remaining patients, we found similar median numbers of conventional radiographs of patients with unilateral and bilateral VND (median 2 vs. 1). On the other hand, we observed that patients with bilateral VND underwent fewer MRI scans compared with patients with unilateral VND (median 0 vs. 2, p = 0.01).

**Figure 4.**
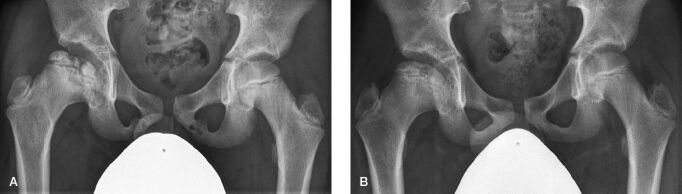
Bilateral VND in a 10-year-old female patient with right-sided Legg–Calvй–Perthes disease (A). Complete dissolution of the bilateral VND at 4-year follow-up (B).

The entire radiographic dissolution of VND was observed in 4 patients, all of which were asymptomatic. In the 1st patient the radiographic dissolution was observed at a 6-month radiographic follow-up examination with a local physician, whilst dissolution in the 2nd patient was observed 3 years following the initial diagnosis as part of the routine oncological follow-up of a Ewing’s sarcoma. The remaining 2 patients both had Legg–Calvé–Perthes disease and bilateral VND. Whilst we observed a synchronous dissolution in 1 patient 4 years after initial diagnosis (Figure 4), the dissolution in the 2nd patient was asynchronous, 2.5 years and 3 years after initial diagnosis, respectively.

**Figure 5.**
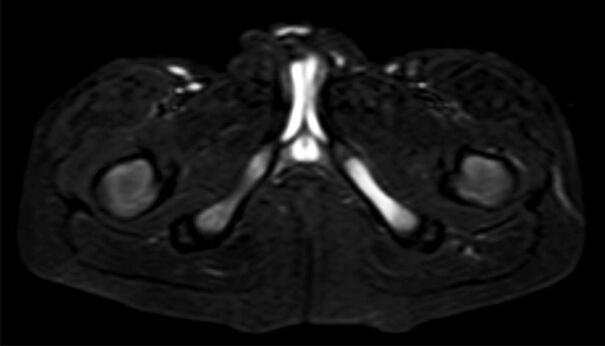
T2-weighted MRI scan with a left-sided VND suspicious of osteomyelitis in a 7-year-old male patient.

A diagnostic biopsy was performed in 2 patients. 1 of them underwent staging for a Ewing’s sarcoma of the right ulna with a fluorodeoxyglucose positron emission tomography/computed tomography (FDG-PET/CT) scan, which showed increased FDG uptake in the left lower pelvis. Despite the fact that conventional radiographs were consistent with the diagnosis of VND, a biopsy was recommended by the treating pediatric oncologists to exclude metastatic disease, given the impact this would have on patient treatment and prognosis. The second patient’s history, laboratory results, and MRI findings were suspicious of osteomyelitis, which could be ruled out in the microbiological and histological exams of the biopsy specimen (Figure 5). While we found regular cortical bone with normal osteoblastic and osteoclastic activity in the first patient, histopathological findings of the second patient showed hyaline cartilage and lamellar bone.

All 15 patients with local complaints reported a resolution of symptoms and return to physical activity after the median follow-up time of 5 months.

## Discussion

In 1923, Odelberg first described “destructive alterations of os ischii” in the adolescent and ruled out trauma, tumor, tuberculosis, and lues as a cause, concluding that “some form of non-specific chronic inflammation” might cause the condition (Odelberg 1923). In 1924, van Neck termed the radiographic observations “osteochondritis of the pubis” (van Neck 1924). Since then, several authors have shown that radiographic changes of the IPS can be part of the normal fusion process during skeletal maturation (Byers 1963, Neitzschman 1997, Ceroni et al. 2004). While in conventional radiographs VND typically presents with a characteristic enlargement or overgrowth of the IPS (Figure 1), hyperintense (T2-weighted) or hypointense (T1-weighted) signals around the IPS are found on MRI (Mixa et al. 2017). However, despite the improved understanding of this physiological process, there is a lack of clinical studies describing the course of VND, and radiographic alterations of the IPS still cause uncertainty among treating physicians.

Our study was designed to address this deficit and found that: (1) the median age at diagnosis of VND is 10 years. (2) Initial diagnosis of VND is more likely to be accurate in asymptomatic than symptomatic patients. (3) More MRI scans are performed in unilateral than in bilateral VND. (4) Nonoperative treatment can quickly lead to a resolution of symptoms and return to full physical activity appears to be possible after a median time of 4 months.

**Figure 6.**
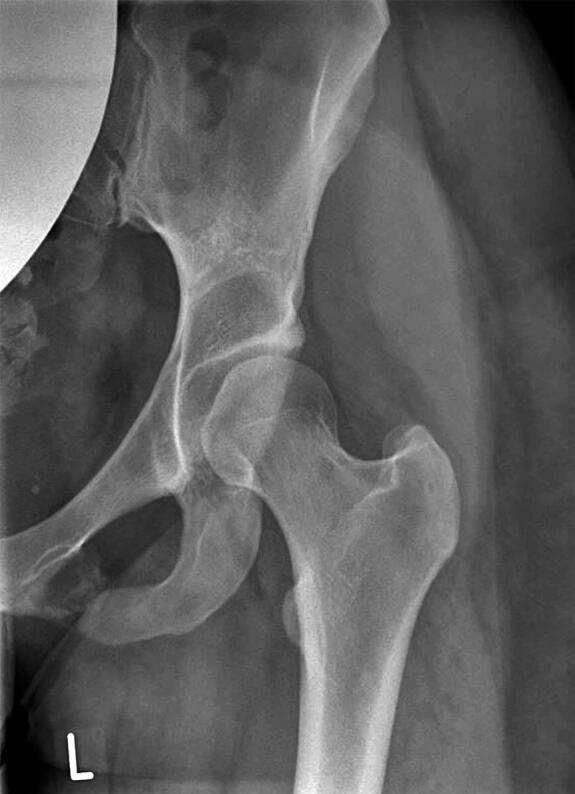
VND in a 22-year-old female patient.

The fusion of the IPS has been shown to be strongly age-related and usually occurs in girls between the age of 4 and 9 years and in boys between the age of 7 and 13 years (Gregory et al. 2019). Although our findings are in general similar, we found a VND in a 22-year-old female patient with a 5-month history of left-sided groin pain and the referral diagnosis of a primary bone tumor (Figure 6). Although the VND in this case was most likely just an incidental finding, this case suggests that not all patients experience complete radiographic resolution of VND after skeletal maturation. Likewise, Morse and Lin (2016) have previously reported a VND case in a 17-year-old female patient with radiographic progression around the IPS during 4-month follow-up leading to a biopsy that confirmed the benign entity. We therefore conclude that physicians should be aware of VND as a possible differential diagnosis in adolescents and young adults, rather than just in prepubertal children.

The synostosis of the IPS can occur simultaneously or asynchronously and asymmetrically and lead to bilateral or unilateral VND, respectively (Cawley et al. 1983). As a bilateral symmetrical appearance is rarely mistaken for pathology, the diagnosis of VND can be especially challenging in unilateral cases (Herneth et al. 2000). This was also the case in our analysis, where all 4 bilateral but only 2 of 17 unilateral cases were initially diagnosed correctly. The most frequent referral diagnosis in our cohort was a suspected primary bone tumor, which is in line with previous reports (Herneth et al. 2000, Mixa et al. 2017). Another possible frequent differential of VND is ischiopubic osteomyelitis (Wait et al. 2011). Wait et al. (2011) have argued that MRI may help to differentiate between the two entities, with ischiopubic osteomyelitis showing typical myositis, abscess, or fluid collections surrounding the IPS whereas VND appears with a characteristic focal area of marrow edema. However, we performed a biopsy in a 7-year-old boy in our cohort with typical laboratory and suggestive MRI findings of osteomyelitis, but the microbiological results were negative and histopathological findings were consistent with VND, where typical histopathology findings include (hyaline) cartilage and lamellar bone (Mixa et al. 2017). The patient underwent nonoperative, non-antibiotic treatment and was asymptomatic after 6 months, highlighting the possible difficulties in diagnosing VND. Another challenging aspect is the diagnosis in patients who suffer from a primary malignant bone tumor, like our patient with Ewing’s sarcoma. A biopsy may sometimes be inevitable to rule out possible bone metastasis, which would impact the further oncological treatment. Similarly, Drubach et al. (2006) have described the case of a 10-year-old girl with a renal cell carcinoma and high uptake around a lucent expansile lesion at the right ischiopubic junction in an FDG PET/CT scan. In a previous scan 3 months earlier the IPS appeared closed and had no pathological uptake, so that the authors performed a biopsy expecting to find metastatic disease, but histopathological analysis confirmed the diagnosis of VND (Drubach et al. 2006).

Patients with VND are frequently subject to regular imaging with ionizing radiation (Cawley et al. 1983, Drubach et al. 2006, Macarini et al. 2011). In a systematic review, Wait et al. (2011) reported 23 radiographs, 17 MRI scans, 4 CT scans, 3 PET/CT scans, and 2 bone scans performed for diagnosis in 29 patients. All of the CT, PET/CT, and bone scans in their study were performed in patients with unilateral VND, a finding we were able to confirm in our cohort (Wait et al. 2011). We also found a higher number of MRI scans being performed in unilateral VND, emphasizing the challenges of diagnosis in unilateral compared with bilateral cases.

As VND is benign self-limiting condition, nonoperative treatment of symptomatic patients is recommended and should be accompanied by clinical and, if necessary, radiographic follow-up examinations (Mixa et al. 2017). However, there are also occasional reports on the surgical treatment of VND (Byers 1963, Oliveira 2010, Wait et al. 2011). Byers (1963) described an excision of the IPS in an 11-year-old boy due to “the severity of pain and uncertainty of the diagnosis” and reported postoperative relief of pain, while Oliveira (2010) performed surgical curettage after failed nonoperative treatment in an 8-year-old boy. Wait et al. (2011) have outlined the lack of long-term follow-up data in VND. Given the results of our study, the resolution of pain in symptomatic patients appears to be very likely under nonoperative treatment, usually within a few months, but symptoms can also last for up to 1 year. A recurrence or possible sequelae was not observed in any of our patients. We therefore strongly recommend avoiding surgery in these patients.

Based on our findings we developed a standardized algorithm for the diagnosis and treatment of VND, in order to reduce uncertainty in treating physicians and avoid unnecessary ionizing radiation imaging in the affected patients (Figure 3).

In conclusion, despite advances in radiographic modalities, VND is still a condition that many physicians find difficult to diagnose. An understanding of the physiological processes of IPS fusion during skeletal maturation is essential and the standardized algorithm we developed may contribute to avoiding unnecessary diagnostic and therapeutic measures and minimizing uncertainty and anxiety amongst affected patients, their families, and treating physicians. Nonoperative treatment appears to be adequate for symptomatic patients and relief of symptoms is usually achieved within a few months, although cases with persisting pain for up to 1 year are possible.

KNS, LPL, GG, CT, MM, RR, BV, and DA designed the study and collected the data. KNS, CT, LPL, and DA were responsible for data management, data analysis, and preparation of figures. KNS and DA wrote the manuscript. KNS, MM, RR, BV, GG, and DA helped with data analysis and with editing of the manuscript.

*Acta* thanks Peter Holmberg Jørgensen and other anonymous reviewers for help with peer review of this study.
